# Introducing the first whole genomes of nationals from the United Arab Emirates

**DOI:** 10.1038/s41598-019-50876-9

**Published:** 2019-10-11

**Authors:** Habiba S. AlSafar, Mariam Al-Ali, Gihan Daw Elbait, Mustafa H. Al-Maini, Dymitr Ruta, Braulio Peramo, Andreas Henschel, Guan K. Tay

**Affiliations:** 1 Center of Biotechnology, Khalifa University of Science and Technology, Abu Dhabi, United Arab Emirates; 2 Department of Biomedical Engineering, Khalifa University of Science and Technology, Abu Dhabi, United Arab Emirates; 3 College of Medicine and Health Sciences, Khalifa University of Science and Technology, Abu Dhabi, United Arab Emirates; 4 Mafraq Hospital, Abu Dhabi, United Arab Emirates; 5 Etisalat-British Telecom Innovation Center, Abu Dhabi, United Arab Emirates; 6 Al Ain Fertility Center, Al Ain, United Arab Emirates; 7 Department of Computer Science, Khalifa University of Science and Technology, Abu Dhabi, United Arab Emirates; 8 School of Psychiatry and Clinical Neurosciences, University of Western Australia, Nedlands, Australia; 9 School of Medical and Health Sciences, Edith Cowan University, Joondalup, Australia

**Keywords:** Structural variation, Structural variation, Genome evolution, Genome evolution, Molecular biology

## Abstract

Whole Genome Sequencing (WGS) provides an in depth description of genome variation. In the era of large-scale population genome projects, the assembly of ethnic-specific genomes combined with mapping human reference genomes of underrepresented populations has improved the understanding of human diversity and disease associations. In this study, for the first time, whole genome sequences of two nationals of the United Arab Emirates (UAE) at >27X coverage are reported. The two Emirati individuals were predominantly of Central/South Asian ancestry. An in-house customized pipeline using BWA, Picard followed by the GATK tools to map the raw data from whole genome sequences of both individuals was used. A total of 3,994,521 variants (3,350,574 Single Nucleotide Polymorphisms (SNPs) and 643,947 indels) were identified for the first individual, the UAE S001 sample. A similar number of variants, 4,031,580 (3,373,501 SNPs and 658,079 indels), were identified for UAE S002. Variants that are associated with diabetes, hypertension, increased cholesterol levels, and obesity were also identified in these individuals. These Whole Genome Sequences has provided a starting point for constructing a UAE reference panel which will lead to improvements in the delivery of precision medicine, quality of life for affected individuals and a reduction in healthcare costs. The information compiled will likely lead to the identification of target genes that could potentially lead to the development of novel therapeutic modalities.

## Introduction

Unified by common cultural practices, religion and language, there are a number of ethnic groups that reside in the region known as the Middle East. A number of geopolitical boundaries group countries into collectives (e.g. the Middle East and North Africa or MENA, the Gulf Cooperative Council or GCC) with common political or economic alliances. In a region that encompasses countries of the Arabian Peninsula (Bahrain, Kuwait, Oman, Qatar, Saudi Arabia and the United Arab Emirates), with North African countries to the west; the Levant to the north and parts of West Asia which includes Iraq and Iran, there are at least nineteen ethnic groups. The Arabs are the largest group and are dispersed across the countries of the Arabian Peninsula, Egypt and Iraq. This group is a subpopulation that primarily speaks the Arabic language, with a number of regional dialects that distinguishes between local subpopulations. The original Arabic language was spoken by populations which descended from ancient Yemeni kingdoms. The language has also largely influenced communities around the Horn of Africa. The language persisted through the development of the D’mt civilization in what is now Ethiopia. These Southern Arabian kingdoms lasted through to the 7th century after which they declined, due to the spread of their Northern counterparts^1^.

To the east of the Arabian Peninsula, a number of different ethnic groups have coexisted together for centuries. The largest of these are the Persians, a group that was established in the first millennium within the Western portion of the Iranian plateau. Approximately 65 percent of the people residing in modern Iran are of this ethnic group^2^ with the remainder comprising a number of different ethnic groups. The Lur are a nomadic people in South-Western Iran. They are mountaineers with close relationships to the Kurds, sharing a similar dialect. The regions in which the Lur reside are co-inhabited by Arabian residence^3^, and as such many are multilingual. There are more Lur people than Arabs in Iran (i.e. 6% vs 2%)^2^. The Lur people are seen as the indigenous Muslims within Iran, who have been least influenced by Western cultures^1^. The Bakhtiyarians are a group that speak a variation of the Lur dialect^1,4^. Kurds are ethnically diverse in comparison to Iranian people due to intermarriages with neighboring ethnic groups^1^. The Kurds were also mountaineers, and like the Lurs, pursued an independent nomadic existence. Kurdish people speak an Iranian branch of the Indo-European language family^1^. At the end of the first world war, the Kurdish people were divided according to boundaries set for Turkey, Iran and Iraq with smaller communities remaining in Syria^5^.

The language spoken by the Baluch is described as belonging to the Indo-Iranian people who mainly reside in the south-eastern region of Iran and across the border into Afghanistan^1^. The Gilaki and Mazandarani reside in Iran and speak a distinct Caspian dialect, rather than Persian^4^. Their languages are described as being closer to a Kurdish relation rather than Persian^1^. Gilaki people are generally found on the Western half of the Caspian southern coastline^4^. Mazandarani people speak a variation of the Caspian dialect as well as Persian^4^. Both groups evident were well established subpopulations that predate rise of the Persian Empire^1^. Talysh people speak a separate variation of the Caspian dialect, Azeri and Persian, but more related to the ancient Medes populations^1^.

The Armenians are a group people who are predominantly Christians that originated in Anatolia, Armenia, now an independent state after the collapse of the Soviet Union^1,6,7^. Assyrians are the indigenous minority group, who continue to identify themselves by their religious, cultural and ancestral backgrounds, from an area that is now Iraq^8,9^.

The influence from Africa to the west of the Middle East include the Beja, a nomadic people within Africa who have lived in Egypt and Sudan for at least 2,000 years^1,4^. The Berber people speak predominantly Arabic and practice the Muslim faith. They are thought to be the closest descendants to the ancient indigenous populations of Africa^10^. A third ethnic group from the west is known as the Copts, a term that was historically used to denote all Egyptians. However, more recently, Copts are used to refer to Christians residing in Egypt^1,11,12^. The Nubians are descendants from distinct ancient Egyptian civilizations^13^ who maintained successful kingdoms from establishing trade routes between Central Africa and the Mediterranean^1^. Due to trade, modern day Nubians slowly acculturated and now share the religious beliefs of Islam with Arabs^13,14^.

In more recent times, the terms, ‘Swahili’ and ‘Zanzibari’ or ‘Zinjibari’, have been used to describe a group of Omani people who have returned from East Africa and Zanzibar after 1964^15–17^. Omani people have had trading outpost in East Africa, predominantly in Zanzibar, since the 17th Century^16^. Consequently, the local Swahili culture around Zanzibar was greatly influenced by the Omani migrants and overseas trade^17^. Swahili became the predominate language of almost all the Omani migrants as well as acceptance to Swahili cultural influences to a point where there is uncertainty as to who were the initial Arabs^16^. The 1964 Zanzibar revolution resulted in Zanzibar gaining independence and the end to slavery at which point, the original Omani Arabs and their families were summoned back to Oman^16^. Some of the Omani who resided on the East coast of Africa intermarried with Africans increasing the heterogeneity of the group^15^. The cultural diversity brought ‘back-from-Africa’ by the Omani people, the African influence including funeral rituals, dress and education continue to persist in Oman^15,16^.

Towards the north of the Arabian peninsula are subpopulations that include the Jews, a religious group mainly residing in Israel with lesser population sizes in Iran, Syria, Lebanon and Egypt^18^. The Turks, another northern ethnic group, are a diverse group of people that inhabit several countries^1^. Turkic rulers initially entered diplomatic relationships with Middle Eastern Empires which later resulted in Turkish conquest that eventually paved the way for the Ottoman Empire^1^. As one of the “longest lived dynasties in global history”, Turkish culture has left its mark on throughout the Middle East^1^.

The Middle East is therefore a truly cosmopolitan part of the world. The entire region sits at the crossroads of significant human migration between the African, European and Asian continents. Mitochondrial DNA (mtDNA) analyses, in particular the D-loop region, has been commonly used for migration studies^19^. Based on these studies, one of the earliest mtDNA lineages (known as the L1 type) is believed to have originated from East Africa around 130,000 years ago, since it is only restricted to Africa^20^. This is the premise for suggesting that the start of ancient genetic migration occurred across Africa and the first wave of human migration out of Africa has been postulated to have occurred approximately 85,000 years ago^19,20^. There are two proposed routes of human migration out of Africa and into the Middle East. The obvious route took place to the north, across the land bridge that is now Egypt and Sinai into the Levantine region^21,22^. The second route was from a location within contemporary Djibouti and Ethiopia across a relatively shallow stretch of water referred to Bab al Mandab Strait into Yemen in the South-Western corner of the Arabian Peninsula^23,24^. The eventual development of trade routes^25^ in more recent history has increased bi-directional gene flow^26^; back into^27^ and out of the region creating the contemporary diversity seen in modern Arabia.

The United Arab Emirates (UAE) sits on the second route out of Africa and was a staging point to Persia, now Iran. Contemporary UAE was formed by the union of 7 emirates or sheikhdoms in 1971 led by Sheikh Zayed bin Sultan Al Nahyan. Of the approximate 10 million population of the UAE, only 10% are citizens of the country. The majority of the residents of the UAE are expatriates, with approximately 30% being South Asian in origin. The genomic organization of UAE nationals has been influenced both by transcontinental migration between Africa, Asia and Europe involving a myriad of different ethnic groups as well as the nomadic lifestyles of some of Arabian populations, particularly the Bedouins. Motivated by the need to understand the origins of the people that live in this South-Western tip of the Peninsula, and their neighbors using genetic data rather than relying only on the ethnolinguistic differences, whole genome sequences (WGS) were completed for two Emiratis. These two WGS are the first ever described for Emiratis and add to other middle-eastern data in the 4 WGS from the Kuwait genome project^28–30^ and 104 WGS from Qatar^31^.

## Results

### Information on subjects and alignment statistics

Two citizens of the United Arab Emirates (UAE) were sequenced in this study. The first (UAE S001) participant was a male aged 87 years. He was diagnosed with hypertension, dyslipidemia, diabetes mellitus and psoriasis. His sample was analyzed using Principal Component Analysis (PCA) and supervised admixture analysis in which all 51 populations from the Human Genome Diversity Project (HGDP) database were used as possible ancestral populations^32^. This analysis showed an admixture ratio of 2.78% (Sub-Saharan Africa), 0.001% (North Africa), 36.96% (Middle East), 54.31% (Central/South Asia), 0.001% (East Asia), 0.001% (Oceania), 5.93% (Europe) and 0.001% (America).

**Figure 1:**
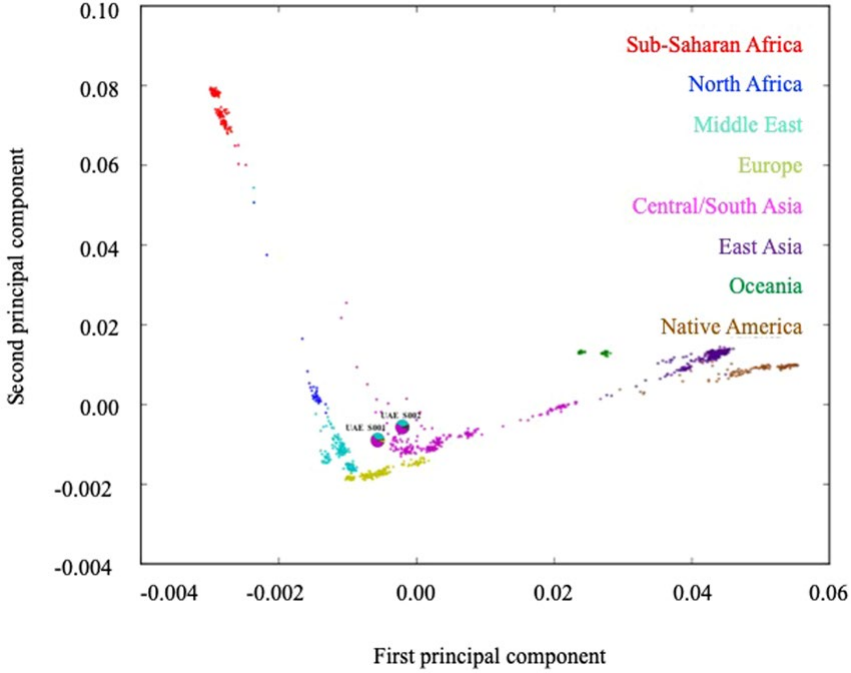
Principal component analysis and supervised admixture analysis representing the estimated ethnic background of UAE S001 and UAE S002 (with admixtrure ratios shown as pie charts) compared to other genotypes of other UAE citizens and those in the HGDP dataset.

The second (UAE S002) sample was of an 87-year old Emirati female, diagnosed with hypertension. Results from the PCA supervised admixture analysis showed an admixture ratio of 3.28% (Sub-Saharan Africa), 2.69% (North Africa), 35.93% (Middle East), 51.31% (Central/South Asia), 2.97% (East Asia), 3.77% (Oceania), 0.001% (Europe) and 0.001% (America). Figure 1 shows the principal components of the admixture ratios of the two Emirati samples as pie charts. These two individuals are shown in the context of genotyping data of other UAE citizens from the Emirates Family Registry and data compiled through the Human Genome Diversity Project (HGDP) that includes individuals of African, Central/South Asian, Eastern Asian, Native American, European and Oceanian descent

Table 1 summarizes the data compiled through the alignment of and genome coverage for the whole genome sequences of UAE S001 and UAE S002. Read lengths of 151 and 152 base pairs (bps) were generated covering the whole genome at 27X and 31X for UAE S001 and UAE S002, respectively. The total number of reads that passed quality control (QC) exceeded 839,000,000 for both individuals. In total, 712,659,088 (83.7%) of reads were mapped or aligned properly to the reference genome, hcg19^33,34^, for UAE S001. The total number of reads mapped to the reference was higher for UAE S002 at 826,900,438 (98.5%). The number of reads mapped in proper pairs was 83.7% and 98.5% in UAE S001 and UAE S002, respectively. There were 857,112 singletons in UAE S001 and 3887,602 in UAE S002.

**Table 1:** Alignment statistics and genome coverage for UAE S001 and UAE S002.

	UAE S001	UAE S002
Number of Reads	851,448,838	839,072,541
Number of Reads Mapped	712,659,088 (83.7%)	826,900,438 (98.5%)
Number of Reads Properly Paired	712,659,088 (83.7%)	826,900,438 (98.5%)
Number of Singletons	857,112 (0.10%)	387,602 (0.05%)
Mean Coverage	27.0909X	31.1866X
Fragment Sizes	151 s	152 s

### Y-chromosome and mitochondrial haplogroups of the participants

The Y haplogroup was determined for UAE S001 using AMY-tree and yHaplo. Both tools indicated that this individual belonged to the y-Haplogroup Q1a2b2 (Q-L933). The Q haplogroup was found to have originated in Central Asia and Southern Siberia, subsequently migrating toward Eurasia, and arriving in the Arabian Peninsula^35–37^.

The mitochondrial haplogroups for the two samples are common in Central/Southern Asia. The R2 + 13500 haplogroup was identified for UAE S001 and the G2a1 haplogroup for UAE S002. The R2 haplogroup is mainly found in Balochistan^38^, and the specific mutation (13500) has been previously identified in Rajasthan and Uttar Pradesh^39^. The G haplogroup is believed to have originated in East Asia, with G2a expanding in Central Asian populations, and subsequently dispersing to neighboring populations^39^.

### Observed single nucleotide polymorphisms and indels

The number of single nucleotide variants for UAE S001 and UAE S002 summarized in Table 2. There was a total of 3,994,521 variants in the first individual, UAE S001, and 4,031,580 variants in UAE S002. The genome-wide (gw) and autosomal (auto) variants in heterozygous and homozygous forms were determined for the two samples. There were 1,646,161 (gw) and 1,560,037 (auto) homozygous as well as 2,348,360 (gw) and 2,343,696 (auto) heterozygous variants in the UAE S001 sample. In the UAE S002 sample, there were 1,576,332 (gw) and 1,527,749 (auto) homozygous as well as 2,455,248 (gw) and 2,387,489 (auto) heterozygous variants (Table 3).

**Table 2:** Summary of variants found in UAE S001 and UAE S002.

		UAE S001	UAE S002
Variants	Total	3,994,521	4,031,580
	‘true’	3,835,491	3,865,759
	‘not listed’	159,030	165,821
SNPs	Total	3,350,574	3,373,501
	‘true’	3,283,240	3,302,437
	‘not listed’	67,334	71,064
Indels	Total	643,947	658,079
	‘true’	552,251	563,322
	‘not listed’	91,696	94,757

**Table 3:** Homozygous and heterozygous (genome-wide vs autosomal) values of the total ‘true’ and ‘not listed’ variants for UAE S001 and UAE S002.

		Type	Homozygous genome-wide	Homozygous autosomal	Heterozygous genome-wide	Heterozygous autosomal
UAE S001	SNPs	Total	1,373,660	1,303,531	1,976,914	1,975,999
		‘true’	1,369,168	1,300,862	1,914,072	1,913,565
		‘not listed’	4,492	2,669	62,842	62,434
	Indels	Total	272,501	256,506	371,446	367,697
		‘true’	240,743	227,983	311,508	311,192
		‘not listed’	31,758	28,523	59,938	59,505
	Total variants		1,646,161	1,560,037	2,348,360	2,343,696
UAE S002	SNPs	Total	1,316,296	1,277,277	2,057,205	2,002,016
		‘true’	1,313,685	1,274,770	1,988,752	1,936,550
		‘not listed’	2,611	2,507	68,453	65,466
	Indels	Total	260,036	250,472	398,043	385,473
		‘true’	230,835	222,856	332,487	322,863
		‘not listed’	29,201	27,616	65,556	62,610
	Total variants		1,576,332	1,527,749	2,455,248	2,387,489

Variants were characterized as ‘true’ and ‘not listed’ if available or missing in the dbSNP 138 database, respectively^40^. Most of the variants identified in UAE S001 and UAE S002 were classified as ‘true’ (96.02% and 95.89%, respectively). Of the total number of variants, the number of Single Nucleotide Polymorphisms (SNPs) and indels in UAE S001 were 3,350,574 (83.88%) and 643,947 (16.12%), respectively. The proportions of SNPs and indels in UAE S002 was similar, at 3,373,501 (83.68%) and 658,079 (16.32%), respectively. Approximately 4% of the total variants identified in the two Emiratis were ‘not listed’; specifically 3.98% for UAE S001 and 4.15% for UAE S002.

The genome-wide and autosomal Transition/Transversion ratios for ‘true’ variants in the two samples are shown in Table 4. The ratios for ‘not listed’ variants were similar: 1.258 (gw) and 1.253 (auto) for UAE S001; and 1.356 (gw) and 1.353 (auto) for UAE S002.

**Table 4:** Transition (Ts) and transversion (Tv) (genome-wide (gw) and autosomal (auto)) values for the ‘true’ and ‘not listed’ variants for UAE S001 and UAE S002.

	Type	Transitions (Ts) genome wide	Transitions (Ts) autosomal	Transversions (Tv) genome wide	Transversions (Tv) autosomal	Ts/Tv gw/auto
UAE S001	‘true’	2,212,013	2,167,261	1,068,928	1,047,128	2.069/2.070
	‘not listed’	37,483	36,198	29,801	28,885	1.258/1.253
	Total	2,249,496	2,203,466	1,098,729	1,076,013	2.047/2.048
UAE S002	‘true’	2,224,741	2,164,997	1,075,220	1,046,288	2.069/2.069
	‘not listed’	40,865	39,075	30,132	28,876	1.356/1.353
	Total	2,265,606	2,204,072	1,105,352	1,075,164	2.050/2.050

^*^Transition: the change of purine (two rings) to purine nucleotide or pyrimidine (one ring) to another pyrimidine; Transversion: the substitution of purine to pyrimidine nucleotide of vice versa.

### Annotation of SNPs and indels

Through the annotation process, variants were classified based on their impact, functional class, and by type within the different genomic locations. These classifications were defined based on SnpEff annotation. Table 5 provides a summary of variants that were categorized into high, low, moderate, and modifiers based on their genomic impact. From UAE S001 and UAE S002 respectively, 99.43% and 99.44% of the total variants were modifiers. The number of total variants with low impact was almost 24 times the number of total variants with high impact in both samples.

**Table 5:** Classification of the ‘true’ and ‘not listed’ genome variants in UAE S001 and UAE S002 samples based on their impact.

		Type	High	Low	Moderate	Modifier
UAE S001	‘true’	Variants	407	11,436	9,463	3,711,873
		SNPs	260	11,436	9,341	3,260,023
		Indels	147	0	122	451,850
	‘not listed’	Variants	91	189	326	143,548
		SNPs	25	189	296	66,762
		Indels	66	0	30	76,786
	Total variants		498	11,625	9,789	3,855,421
UAE S002	‘true’	Variants	400	11,561	9,392	3,739,005
		SNPs	262	11,561	9,269	3,278,963
		Indels	138	0	123	460,042
	‘not listed’	Variants	79	233	380	149,511
		SNPs	24	233	250	70,405
		Indels	55	0	30	79,106
	Total variants		479	11,794	9,772	3,888,516

Table 6 presents variants of the two genomes classified into four functional classes. The number of total variants of each functional class (missense, nonsense, silent, or none identified) for UAE S001 was similar to that of UAE S002. Tables 7 and 8 are summaries of variants classified into 23 groups according to genomic location. Furthermore, the two tables summarizes (in brackets) the number of “real” and “not listed” variants that overlap with poorly-resolved regions or low complexity regions, which includes segmental duplications, rDNA chromosome arms, centromeric, telomeric, large retrotransposable elements, etcetera as provided by UCSC Table Browser^41^ for samples UAE S001 and UAE S002. Most of the ‘true’ and ‘not listed’ variants lie in intergenic regions (52.58% of the total variants for UAE S001, and 52.71% of the total variants for UAE S002), followed by those that lie in the introns. It is also worth noting that >50% of the SNPs and >68% of the indels that are intergenic variants are located in the low complexity regions. Table 9 summarizes the variants of UAE S001 and UAE S002 (listed or not) with respect to GnomAD, showing a significant increase in the true variants in comparison to dbSNP 138. Additionally, Table 10 is a summary of the genic variants that are not listed with respect to GnomAD for both samples.

**Table 6:** Classification of the ‘true’ and ‘not listed’ genome variants in the UAE S001 and UAE S002 samples based on their functional class.

		Type	Missense	Nonsense	Silent	None
UAE S001	‘true’	Variants	9,388	70	10,604	3,713,117
		SNPs	9,388	70	10,604	3,260,998
		Indels	0	0	0	452,119
	‘not listed’	Variants	296	14	164	143,680
		SNPs	296	14	164	66,798
		Indels	0	0	0	76,882
	Total variants		9,684	84	10,768	3,856,797
UAE S002	‘true’	Variants	9,316	63	10,734	3,740,245
		SNPs	9,316	63	10,734	3,279,942
		Indels	0	0	0	460,303
	‘not listed’	Variants	352	12	206	149,633
		SNPs	352	12	206	70,442
		Indels	0	0	0	79,191
	Total variants		9,668	75	10,940	3,889,878

^*^SnpEff assigns a functional class to certain effects, in addition to an impact: Nonsense: assigned to point mutations that result in the creation of a new stop codon; Missense: assigned to point mutations that result in an amino acid change, but not a new stop codon; Silent: assigned to point mutations that result in a codon change, but not an amino acid change or new stop codon; None: assigned to all effects that don’t fall into any of the above categories (including all events larger than a point mutation).

**Table 7:** Summary of the ‘true’ and ‘not listed’ genome variants for UAE S001 classified by type within the different genomic locations.

TYPE	Total (UAE S001)	‘true’ (UAE S001)		‘not listed’ (UAE S001)	
	Variants	SNPs	Indels	SNPs	Indels
Codon change plus codon deletion	54	0	46 (24)	0	8 (2)
Codon change plus codon insertion	26	0	20 (9)	0	6 (5)
Codon deletion	23	0	17 (8)	0	6 (1)
Codon insertion	49	0	39 (8)	0	10 (7)
Downstream	149,159	119,500 (58,265)	22,970 (14,553)	2,806 (1,242)	3,883 (3,035)
Exon	6,560	5,860 (2,478)	475 (251)	149 (48)	76 (55)
Frameshift	154	0	96 (22)	0	58 (17)
Intergenic	2,038,588	1,686,497 (950,612)	273,365 (187,586)	32,321 (17,386)	46,405 (38,390)
Intragenic	3	0	3 (2)	0	0
Intron	1,448,583	1,276,103 (598,957)	124,733 (76,992)	26,576 (12,380)	21,171 (16,691)
Nonsynonymous coding	9,637	9,341 (681)	0	296 (28)	0
Nonsynonymous start	1	1	0	0	0
Splice site acceptor	101	50 (12)	40 (5)	6	5 (2)
Splice site donor	112	94 (23)	11 (2)	5	2
Start gained	856	831 (114)	0	25	0
Start lost	22	22 (1)	0	0	0
Stop gained	84	70 (7)	0	14 (1)	0
Stop lost	25	24	0	0	1
Synonymous coding	10,761	10,597 (493)	0	164 (19)	0
Synonymous stop	7	7	0	0	0
Upstream	117,150	141,568 (66,745)	26,836 (17001)	3,975 (1,691)	4,771 (3,718)
Untranslated 3′	29,579	25,470 (5,244)	3,040 (1,066)	688 (135)	381 (207)
Untranslated 5′	5,799	5,025 (654)	428 (120)	247 (55)	99 (55)

The numbers in brackets reflect the number of those variants located in poorly resolved regions (i.e. low complexity regions such as segmental duplications, rDNA chromosome arms, centromeric, telomeric, large retro-transposable elements and others that are provided by the UCSC Table Browser).

**Table 8:** Summary of the ‘true’ and ‘not listed’ genome variants for UAE S002 classified by type within the different genomic locations.

TYPE	Total (UAE S002)	‘true’ (UAE S002)		‘not listed’ (UAE S002)	
	Variants	SNPs	Indels	SNPs	Indels
Codon change plus codon deletion	56	0	48 (26)	0	8 (4)
Codon change plus codon insertion	30	0	21 (7)	0	9 (3)
Codon deletion	25	0	20 (12)	0	5 (4)
Codon insertion	42	0	34 (9)	0	8 (7)
Downstream	148,446	118,309 (56,772)	23,306 (14,930)	2,993 (1,284)	3,838 (3,108)
Exon	6,601	5,844 (2,450)	496 (268)	162 (47)	100 (61)
Frameshift	126	0	82 (24)	0	44 (15)
Intergenic	2,061,266	1,699,950 (953,338)	279,427 (191,786)	33,928 (17,788)	47,961 (40,218)
Intragenic	2	0	2 (1)	0	0
Intron	1,459,683	1,283,402 (598,698)	126,283 (78,210)	28,294 (12,623)	21,704 (16,945)
Nonsynonymous coding	9,620	9,270 (633)	0	350 (21)	0
Nonsynonymous start	1	1	0	0	0
Splice site acceptor	115	59 (8)	45 (8)	7	4
Splice site donor	114	94 (19)	10 (1)	3	7 (4)
Start gained	854	827 (109)	0	27 (4)	0
Start lost	24	22	0	2	0
Stop gained	76	63 (6)	1	12 (2)	0
Stop lost	24	24 (1)	0	0	0
Synonymous coding	10,935	10,729 (472)	0	206 (19)	0
Synonymous stop	6	6	0	0	0
Upstream	176,928	140,927 (65,564)	27,030 (17,176)	3,993 (1,591)	4,978 (3,965)
Untranslated 3′	29,851	25,599 (5,252)	3,045 (1,090)	807 (145)	400 (215)
Untranslated 5′	5,739	4,932 (649)	453 (134)	229 (49)	125 (68)

The numbers in brackets reflect the number of those variants located in poorly resolved regions (i.e. low complexity regions such as segmental duplications, rDNA chromosome arms, centromeric, telomeric, large retro-transposable elements and others that are provided by the UCSC Table Browser).

**Table 9:** Summary of listed or unlisted variants (with respect to GnomAD) for the UAE S001 and UAE S002, showing a significant increase in the true variants in comparison to dbSNP 138.

TYPE	UAE S001					UAE S002				
	Total	‘true’		‘not listed’		Total	‘true’		‘not listed’	
	Variants	SNPs	Indels	SNPs	Indels	Variants	SNPs	Indels	SNPs	Indels
Codon change plus codon deletion	54	0	52	0	2	56	0	53	0	3
Codon change plus codon insertion	26	0	25	0	1	30	0	28	0	2
Codon deletion	23	0	21	0	2	25	0	25	0	0
Codon insertion	49	0	48	0	1	42	0	40	0	2
Downstream	149,159	121,377	25,819	929	1,034	148,446	120,336	26,079	966	1,065
Exon	6,560	5,966	539	43	12	6,601	5,965	579	40	17
Frameshift	154	0	143	0	11	126	0	117	0	9
Intergenic	2,038,588	1,709,914	307,629	8,904	12,141	2,062,522	1,725,073	315,180	9,339	12,930
Intragenic	3	0	3	0	0	2	0	2	0	0
Intron	1,448,583	1,294,273	140,543	8,406	5,361	1,459,726	1,303,159	142,225	8,569	5,773
Nonsynonymous coding	9,637	9,503	0	134	0	9,620	9,489	0	131	0
Nonsynonymous start	1	1	0	0	0	1	1	0	0	0
Splice site acceptor	101	53	45	3	0	115	61	49	5	0
Splice site donor	112	97	13	2	0	114	95	17	2	0
Start gained	856	849	0	7	0	854	851	0	3	0
Start lost	22	22	0	0	0	24	24	0	0	0
Stop gained	84	78	0	6	0	76	70	1	5	0
Stop lost	25	24	0	0	1	24	24	0	0	0
Synonymous coding	10,761	10,693	0	68	0	10,935	10,854	0	81	0
Synonymous stop	7	7	0	0	0	6	6	0	0	0
Upstream	117,150	144,105	30,293	1,438	1,314	176,930	143,564	30,586	1,358	1,422
Untranslated 3′	29,579	25,885	3,340	273	81	29,874	26,164	3,355	263	92
Untranslated 5′	5,799	5,167	513	105	14	5,742	5,074	553	90	25

**Table 10:** Summary of the variants that are ‘not listed’ (with respect to GnomAD) for UAE S001 and UAE S002.

Type	UAE S001			UAE S002		
	Total	SNPs	Indels	Total	SNPS	Indels
Frameshift	11	0	11	9	0	9
Exon	55	43	12	57	40	17
Codon change plus codon deletion	2	0	2	3	0	3
Codon change plus codon insertion	1	0	1	2	0	2
Codon deletion	2	0	2	0	0	0
Codon deletion	2	0	2	0	0	0
Intron	13,767	8,406	5,361	14,342	8,569	5,773
Nonsynonymous coding	134	134	0	131	131	0
Splice site acceptor	3	3	0	5	5	0
Splice site donor	2	2	0	2	2	0
Synonymous coding	68	68	0	81	81	0
Synonymous stop	0	0	0	0	0	0
Untranslated 3 prime	354	273	81	355	263	92
Untranslated 5 prime	119	105	14	115	90	25
Total	14,520			15,102		

### Variants associated with specific diseases

**Figure 2:**
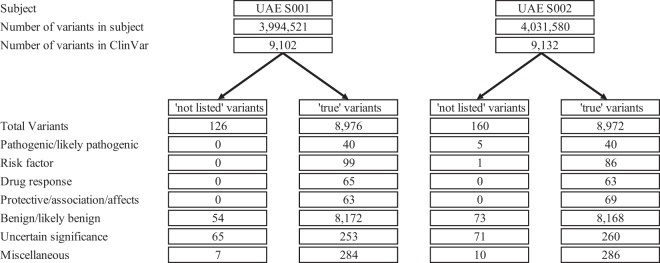
A pipeline chart showing the number and types of variants in the UAE S001 and UAE S002 samples.

It is important to delineate the genotype-disease association for personal genomes by relating the variants to potential susceptibility for certain disorders. The 23 genomic classes were further annotated according to the clinical significance of the variant (pathogenic, likely pathogenic, drug-response, risk-factor, affection, and association) with reference to the ClinVar and OMIM databases (Table S1). Figure 2 shows the clinical significance classification based on the databases used and the number of variants identified in each class for the two UAE participants.

### Concordance in SNP calls between the deep sequencing experiment and genotyping experiment using Bead Chip array

Next Generation Sequencing (NGS) results for the UAE S001 sample were compared to genotyping data obtained for the subject using the Illumina Omni 5 Exome bead chip technology. After applying quality control, the intersection of the remaining SNP positions and the single nucleotide variant calls from UAE S001 NGS yielded 226,007 SNPs. Of these, 275 (or 0.12%) were not concordant. Similarly for UAE S002, the comparison of NGS and array data yielded 160,608 SNPs. Of these, 111 (or 0.069%) were not concordant.

### Comparing the sequenced genomes with individual genomes from other continents

**Figure 3:**
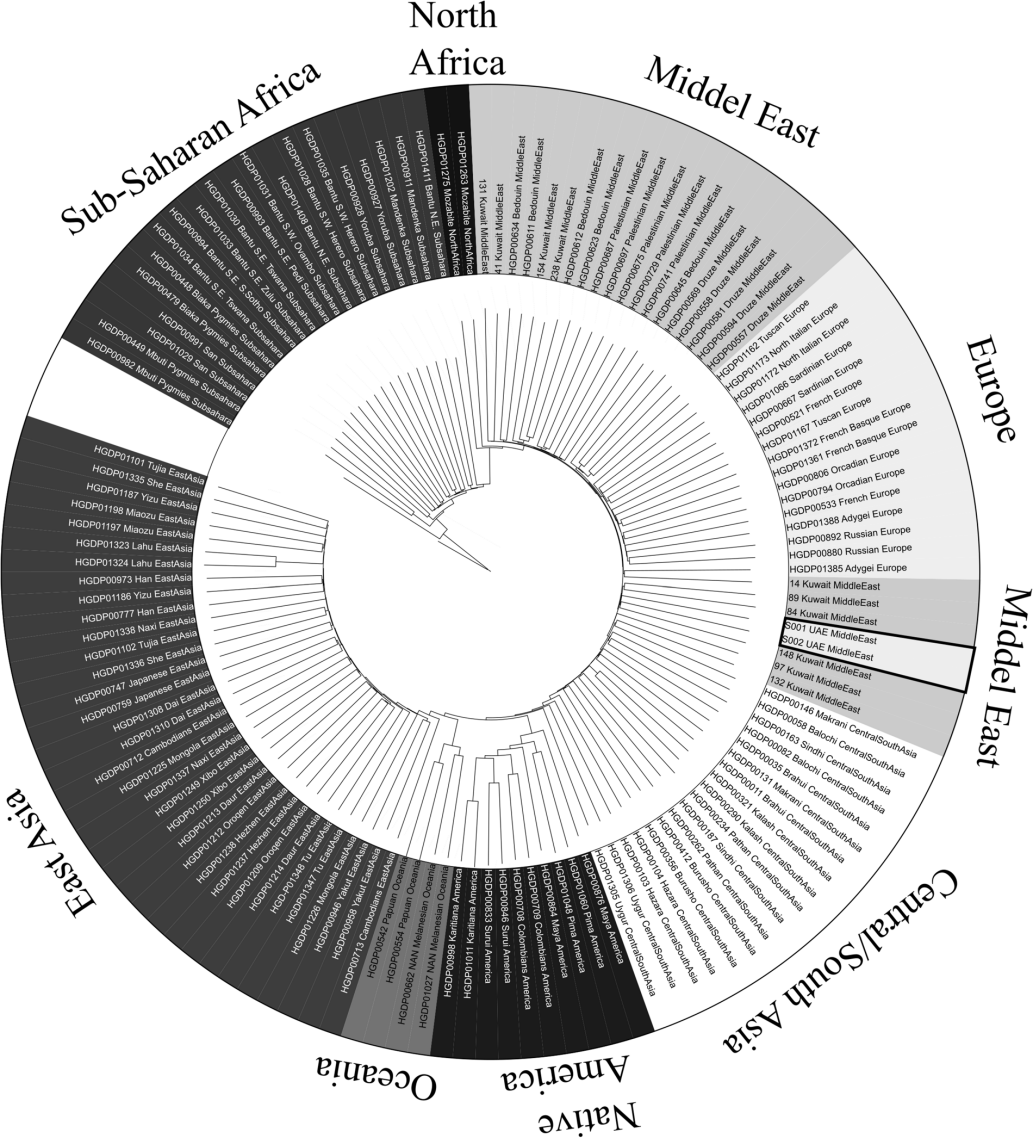
Intergenome distances between genomes of UAE S001, UAE S002, Kuwaiti and individuals from the 51 populations in the HGDP.

A phylogenetic tree comparing subjects UAE S001 and UAE S002 with Human Genome Diversity Project (HGDP) and additional available data of Kuwaiti genome^29,42^ was constructed using the neighbor-joining method and shown in Figure 3. The two local samples cluster with genome data from the Kuwaiti study and near the population representing Central/ South Asia. All populations fall into respective clades. However, European Middle Eastern subjects fall into the same cluster. The fact that they are not in entirely separated subclades can possibly be attributed to limited number of common variants available for analysis, with only 20,658 common variants used.

**Figure 4:**
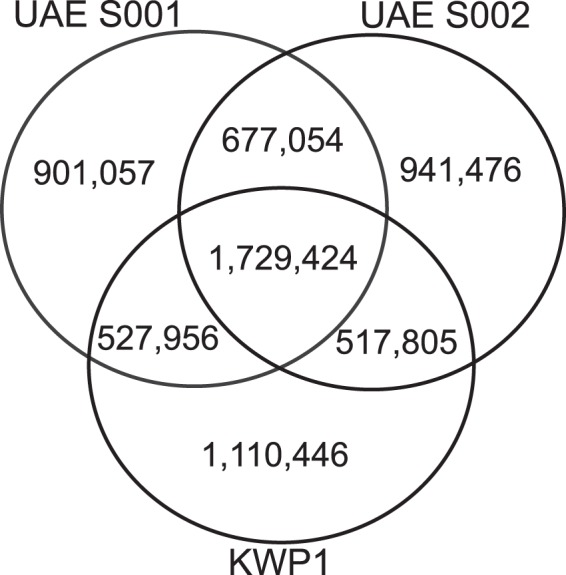
Venn diagram presents the intersections of known variants among UAE S001, UAE S002 and KWP1 (individual of Persian ancestry from Kuwait).

Further, the number of variations identified for both UAE S001 (3,994,521) and UAE S002 (4,031,580) genomes was comparability higher than the total number identified from a whole genome sequence of an Indian individual^43^ of around 3.4 million, when aligned to hg19. Additionally, it was slightly higher than seen in the sequenced individual (3,977,914) from the Persian subgroup of Kuwaiti population (KWP1)^28^. Figure 4 shows a Venn diagram of the total identified variants in the two UAE samples and KWP1 in which 1,729,424 variants were found in the three samples.

## Discussion

There is an intolerable gap in the human genome landscape. Despite the best efforts of the Human Genome Organization (HUGO), Haplotype Map (HapMap) and other international consortia, genome data from ethnic groups of the Arab-speaking world is underrepresented. In a recent audit of genome data in the public domain, genome data from populations of the Middle East was less than 1%^44^.

Here, the whole genome sequence of two Emiratis using next-generation sequencing (NGS) technology is presented. We report around four million genome variants, some of which are ‘not listed’ in dbSNP 138 dataset. Furthermore, to determine the actual continental or population contributions for the two studied samples, ADMIXTURE was run in supervised mode with reference populations from HGDP. Figure 1 shows principal component analysis supervised admixture for the two samples showing both have contributions from Central/South Asian populations.

The Y-chromosome haplogroup (Q1a2b2 (Q-L933)) for the male sample (UAE S001) is consistent with the individual with origins from Central/Southern Asia. Furthermore, the mitochondrial DNA lineages of both individuals also indicate a maternal line from Central/Southern Asia regions.

The whole genome of the two Emirati samples was sequenced at a coverage depth of greater than 27X. The distributions of variants were almost the same in the two Emiratis when compared with the human reference genome (hg19)^33,34^. This included homozygous variants (41.21% (gw), 39.05% (auto)), heterozygous variants (58.79% (gw), 58.67% (auto)) in UAE S001. There were 39.10% (gw) and 37.89%(auto) homozygous variants as well as 60.90% (gw) and 59.21% (auto) heterozygous variants in UAE S002. These proportions of homozygosity/heterozygosity were almost in concordance with the proportions in sequencing 100 Malay Genomes using Next Generation Sequencing (NGS)^45^.

SNPs and indels were checked against the dbSNP 138 database^40^. Up to 96% of the SNPs that were identified were classified as ‘true’. Of the total number of variants in UAE S001, 16.12% were indels. The proportion of indels in UAE S002 was also similar, at 16.32%. Approximately 4% of the total variants identified in the two Emiratis were ‘not listed’: 3.98% for UAE S001 and 4.15% for UAE S002. Novel variants were as low as 0.01% when compared to GnomAD. Most of the ‘true’ and ‘not listed’ variants were localized to intergenic regions (52.58% of the total variants for UAE S001 and 52.71% of the total variants for UAE S002), followed by those that were in introns. It is also worth noting that >50% of the SNPs and >68% of the indels that were intergenic in nature were found in the low complexity regions (Tables 7 and 8**)**. This is consistent with the observations made in a Kuwaiti study of a Bedouin subgroup (KWB)^29^ using Illumina technology for whole genome sequencing. Of all the variants in UAE S001, 33.60% were in the intronic region. Similarly for UAE S002, 33.54% of the variants were in the intronic region. Of the coding variants, 70 ‘true’ variants were identified as stop-gained and 24 ‘true’ variants as stop-lost in UAE S001. In the UAE S002 sequence, 64 ‘true’ coding variants were identified as stop-gained and 24 ‘true’ variants as stop-lost. These variants can elongate or truncate the coded protein sequence.

There number of true variants with high impact on protein coding process in UAE S001 included 70 nonsense and 24 missense variants. In UAE S002, there were 63 nonsense and 24 missense variants. In addition, among the total coding variants identified as stop-gained or stop-lost, 14 in UAE S001 and 12 in UAE S002 were ‘not listed’ variants. Moreover, ‘true’ variants identified with loss of function (LOF) from the coding regions in UAE S001 and UAE S002 were categorized and is presented in Table S2. A set of 467 protein coding variants (384 ‘true’ variants and 83 ‘not listed’ variants) were annotated as loss of function in UAE S001. There were 451 loss of function variants (376 ‘true’ variants and 75 ‘not listed’ variants) in UAE S002. Two hundred and nineteen variants in UAE S001 and 220 variants in UAE S002 were homozygous leading to complete loss of function. Of the annotated variants that were ‘true’ to have loss of function, the majority were identified in the splice site regions (119 in UAE S001, and 130 in UAE S002) followed by frame shifts region (75 in UAE S001, and 66 in UAE S002). On the other hand, only 2 homozygous modifier insertions were identified in the third prime untranslated region UTR 3’ in each of the genomic sequences.

For the identification of novel and known variants in the two samples, the dbSNP 138 version where novel and known indicates whether the variant was ‘true’ or ‘not listed’ was used^40^. Since more recent databases such as dbSNP 151^40^ and the GnomAD^46^ database are now available, these were used as the basis for identifying those variants that are novel. For example, the called variants from both UAE S001 sample and UAE S002 sample that were found to be listed in the dbSNP 151 were significantly less than the ‘not listed’ variants reported. For UAE S001 it changed from 159,030 variants in dbSNP 138 to 55,489 variants in dbSNP 151; and for UAE S002 it changed from 165,821 variants in dbSNP 138 to 57,734 variants in dbSNP 151. Additionally, when compared with GnomAD, the number of the variants decreased further (GnomAD for UAE S001: 45,087 variants; GnomAD for UAE S002: 47,339 variants) resulting in only around 28.35% and 28.5% of the ‘not listed’ variants for UAE S001 and UAE S002 respectively, being called “novel” variants (not reported in GnomAD). This indicates that the previously “not listed” variants called were indeed genuine variants as they were subsequently identified in GnomAD, part of which is classified by type within the different genomic locations as reported in Table 9. When the regions with genes for the two genomes were compared with variants in GnomAD variants, 14,520 variants for UAE S001 and 15,102 variants for UAE S002 were obtained and listed in Table 10.

The Transition/Transversion (Ti/Tv) ratio is usually used as a quality measure for called variants and is calculated for both genome-wide and autosomal variants (Table 4**)**. The ‘true’ variants was 2.069 for both individuals which were in agreement with the expected range of 2.0 to 2.1 for whole genome sequencing^47^. The values for ‘not listed’ variants were 1.258 and 1.356 for UAE S001 and UAE S002 respectively, which is lower than the expected ratio of 2. This could be due to the fact that in the variant calling pipeline the VQSR target truth sensitivity was set at 99.9, which could have been excessively stringent. According to Cai *et al*. (2017) a sensitivity VQSR target truth of 90 was found to optimize the balance of the Ti/Tv ratio of the novel variants with retaining as many potential novel variants as possible^48^. Therefore, the data was reanalysed using the lenient VQSR target truth sensitivity of 90. The Ti/Tv ratio of the ‘not listed’ variants indeed increased to 1.619 and 1.88 for UAE S001 and UAE S002 respectively. Other reasons for the low ratio could include one or a combination of different factors which include sequencing errors resulting in residual false positives, a relative deficit in transitions due to sequencing context bias, or a higher transition ratio that can result from low frequency variants^49^. Furthermore, the autosomal values were found to be, as expected, less than the genome-wide variants but the Ts/Tv ratios were not significantly different.

In this study, several methods were used to estimate the genetic ancestry to understand the admixture of the two samples that were chosen from the UAE population for this study. The two samples were not chosen to represent all ethnic groups of the UAE population. Principal Component Analyses were performed on both UAE S001 and UAE S002 genomes to estimate their ethnic composition by correlating their genetic polymorphisms with data of different populations in the HGDP. The principal component based method is the most commonly used method for many large dense genotype datasets^50^. The results of the genetic ancestry analysis illustrate the different ethnic background of the two individuals with a influence from the Central/Southern region of Asia.

Genetic ancestry can also be deduced from mtDNA and Y chromosome haplogroups or by using multiple unlinked autosomal markers^51^. To confirm the genealogical ancestor of the UAE S001 sample, the Y-chromosome Haplogroup was determined using AMY-tree and yHaplo. The Q1a2b2 (Q-L933) Haplogroup for the male subject, UAE S001 is a member of the Q Haplogroup, which mostly frequent among the Amerind^35^. However, a study of 471 individuals with subclades of the Q haplogroup by Huang *et al*. (2018) concluded that the Q haplogroup originated from Central Asia and Southern Siberia and dispersed to the Amerind and subsequently to whole Eurasia and part of Africa^37^. The Q haplogroup was found to have arrived in the Arab Gulf region, across Iran, from central Southern and Southeast Asia and were found to be abundant in the UAE, Iran and Pakistan^36^.

Mitochondrial (mtDNA) haplogroups were determined for both samples using Haplogrep. The R2 + 13500 haplogroup was identified in UAE S001, a lineage which is mostly concentrated in Southern Pakistan and India^38,52^. A study that focused on the human mtDNA variation in the Southern Arabia identified the presence of the R2 clade in Arabia and nearby regions^53^. As for UAE S002 sample, the G2a1 haplogroup that was identified is a lineage found mainly in Central Asia, with some overflow at low frequencies in adjacent regions including Iran and Southwest Asia^54^.

The extent of variability in the two Emirati genomes, UAE S001 and UAE S002, were determined by comparison to genomes from different world population. The two Emirati genomes cluster with a Kuwaiti genome. Additionally, both Emirati genomes clustered with the Central Asian group in reference to the HGDP dataset on the phylogenetic tree (Fig. 3), which is consistent with the rest of the analyses performed here. As elucidated earlier, migration and population movement were common events that widely occurred throughout the region spanning from Southern Asia across the Levantine and the Arabian Peninsula to North Africa, confirming the likelihood of the admixtures found in the 2 genomes that were studied.

Disease susceptibility and many inherited traits are affected by interactions between different variants located in multiple genes spread across the genome^55^. A total of 213 variants were identified in the splice site acceptor and splice site donor regions in UAE S001; with three variants of clinical significance. These include a known homozygous SNP (rs2004640) in *IRF5* gene that has been shown to be associated with Rheumatoid Arthritis, a heterozygous deletion (rs1799759) in the *A2M* gene that is a risk factor for the susceptibility to Alzheimer’s disease, and a heterozygous SNP (rs10774671) known to result in the loss of function of the *OAS1* gene, a high impact risk factor for susceptibility to Type 1 Diabetes. As for UAE S002, 209 variants were identified in the splice site acceptor and splice site donor regions, in which only one is clinically significant. The heterozygote SNP (rs10774671) is known to cause loss of function in the OAS1 gene and is a high impact risk factor for susceptibility to Type 1 Diabetes.

Sixty-nine variants in the intronic region in the sequence data of UAE S001 may have specific clinical relevance to the individual’s reported medical history, such as diabetes, obesity and cholesterol. For example, two genotypes linked with the susceptibility of Type 2 Diabetes Mellitus (T2DM); rs7903146 SNP in the *TCF7L2* gene [OMIM: 125853], a heterozygous modifier affecting drug response, and the rs4402960 SNP in *IGF2BP2* gene [OMIM: 125853] a heterozygous risk factor modifier. Two other heterozygous genotypes in the *WFS1* gene (rs10010131 SNP and rs6446482 pathogenic SNPs) have also previously been shown to be associated with Type 2 Diabetes Mellitus. An obesity linked protein coding variant rs1421085 in the *FTO* gene has previously been defined as a heterozygous risk factor modifier. A heterozygous protein coding rs326 variant in the *LPL* gene is a modifier known to be associated with high density lipoprotein cholesterol level quantitative trait locus 11. As for UAE S002, two heterozygous risk factors were related to the susceptibility of Type 2 Diabetes Mellitus, specifically rs3792267 [OMIM:125853] and rs4402960 [OMIM:125853]. Another two heterozygous variants were associated with Non-insulin Dependent Diabetes Mellitus located within the *WFS1* gene; rs10010131 and rs6446482.

They were six clinically significant variants in the downstream region of the UAE S001 whole genome sequence. Of these, only two were of particular interest as they were heterozygous risk factors of Type 2 Diabetes Mellitus. Both rs11196205 [OMIM:125853] and rs122555372 [OMIM:125853] are variants located in TCF7L2 gene, that has been widely studied as a marker for Type 2 Diabetes Mellitus.

There were 84 non-synonymous coding variants with missense function in the whole genome sequence of the UAE S001 participant. Of these, four variants were associated with Type 1 Diabetes (rs2476601, rs231775, rs237025, rs1131454), two with Maturity Onset Diabetes of the Young (rs5219, rs1169288), two with Type 2 Diabetes Mellitus (rs13266634, rs5219), and two with microvascular complications of diabetes (rs4880, rs854560). Moreover, three cholesterol related variants were identified: rs6180 variant in the *GHR* gene [OMIM:143890], a heterozygous risk factor for familial hypercholesterolemia; rs5370 variant in the *EDN1* gene identified with heterozygous association with High Density Lipoprotein (HDL) cholesterol levels; and rs5882 variant in the *CETP* gene [OMIM:143470], a heterozygous SNP associated with Hyperalphalipoproteinemia. Additionally, the variant rs1042714 located in the *ADRB2* gene was identified as a risk factor for obesity with moderate impact. Another locus of particular interest was rs33980500 [OMIM: 614070] in the *TRAF3IP2* gene as it has been identified as a risk factor for Psoriasis, a skin related condition. A hypertension related variant was also identified as a protein coding risk factor residing in the *NOS3* gene. Upon closer inspection of the whole genome sequence data of UAE S002, genetic variants related to diabetes, hypertension, cholesterol and obesity related were present. In particular, two hypertension related mutations were identified; a homozygous risk factor rs699 locus was found to have a missense functional class causing an amino acid change (M268T) and a heterozygous risk factor rs1799983 locus in the *NOS3* gene casing an amino acid change (D298E).

It is important to note that these genetic variations alone do not provide definitive diagnosis of a specific disorder. It is challenging process to describe the genetic underpinnings and the genome architecture of common complex traits and multifactorial chronic diseases as these are influenced by multiple loci and genetic factors^56^, with contribution from the environment. Nevertheless, sequencing of whole genomes in the UAE will continue as it will give access to all, including ‘true’ and ‘not listed’ variants, which can be used to initiate functional studies to identify the contribution of casual variants to human phenotypes^57^.

This study is a step that adds to the efforts in neighboring countries to address the deficiency in genomic data on populations of the Middle East. Importantly, a review of the literature in the PubMed and Science Direct databases has revealed a lack of information in the UAE. Despite smaller populations in Qatar and Kuwait, whole genome sequences are available^28,31^. However, there have been no studies published on the whole genome sequence of the UAE population. Therefore, this presentation of the first ever whole genome sequence in the UAE is important as it is expected to lead to greater initiatives in genome-based medicine including improved understanding of chronic disease among its populous and the development of new paradigms in medicine, specifically the establishment of precision, personalized and P4-type strategies^58^.

## Materials and Methods

### Sample and DNA extraction

Prior to enrolment, the two subjects (UAE S001 and UAE S002) provided their written informed consent on a form that had been approved by the Institutional Ethics Committee IRB (Institute Review Board) of Mafraq Hospital in Abu Dhabi, United Arab Emirates (UAE). All experimental protocols were approved by the IRB of Mafraq Hospital in Abu Dhabi and all methods were performed in accordance with the guidelines and regulations of this IRB.

Subjects were also given a questionnaire to collect their historical and demographical information. To be included in the study, subjects had to be an adult (>18 years old) citizen of the UAE who understood their contribution to the study and was subsequently able to give consent.

Saliva samples were collected from the two subjects using the Oragene OGR-500 kit (DNA Genotek, Ottawa, Canada). The prepIT®L2P system (DNA Genotek, Ottawa, Canada) was used to extract genomic DNA from buccal cells in the saliva samples. The extracted DNA aliquots were quantified using the DS-11 FX Fluorometer (Denovix Inc. Wilmington DE, USA) and the integrity of each was checked by electrophoresis on an agarose gel.

### Library preparation

Libraries for each individual were prepared from the cleaned and sheared genomic DNA (gDNA) using the protocol provided and recommended by the manufacturer of the Illumina TruSeq® DNA PCR-Free Library Prep kit (Illumina Inc., San Diego CA, USA). The indexed paired-end libraries were then quantified using the Denovix DS-11 FX Fluorometer and sizes were confirmed using the Advanced Analytical Fragment Analyzer (Advanced Analytical Technologies Inc., Ankeny IA, USA). The Kapa Library Quantification Kit for Illumina platforms (ROX low qPCR mix) (Kapa Biosystems, Wilmington MA, USA) was used to quantify the NGS indexed pair-end libraries that were loaded into a ViiA 7 Real-Time PCR system (Thermo Fisher Scientific, Waltham MA, USA) to determine the optimal loading concentration of gDNA, providing the adequate clustering density on the flow cell during library sequencing. The libraries were loaded into NextSeq. 500 (Illumina Inc., San Diego CA, USA) separately, for paired-end sequencing using a setting that at least 75% of the bases will be called with a quality score >Q30.

### Alignment of reads from whole genome sequencing

Alignment results were generated for the raw reads of the two Emiratis samples (UAE S001 and UAE S002) using BWA v0.7.12^59^ (BWA-MEM) by mapping raw reads to the human reference genome hg19^33,34^ with reads of 151 base-pairs (bps) in length.

### Single Nucleotide Polymorphism (SNP) and indel discovery

The Picard v2.9.4^60^, Genome Analysis Toolkit (GATK) v3.7^61^ and Qualimap software version 2.2.1 were used for the processing and quality control of the aligned files (BAM) before the process of variant calling.

Haplotypes were identified using GATK HaplotypeCaller, a tool that performs local reassembly, calls the variants, and subsequently outputs a VCF (Variant Call Format) file of variants classified into SNPs and indels. According to GATK best practice, additional Variant Quality Score Recalibration (VQSR) and filtration steps were performed on the VCF file^62,63^. A 2-stage VQSR process was performed using the GATK VariantRecalibrator tool and the ApplyRecalibration tool which were used for SNPs recalibration and indel recalibration separately. Classes of polymorphisms; SNPs and indels; were assessed and scored based on a standard Gaussian mixture model while using highly validated variant resources (hapmap, 1000 G, Omni, dbSNP 138).

### SNP annotation method

The variants that were catalogued in the VCF file format were annotated using the genomic annotation tool SnpEff version 3.4^64^. This tool was developed with predictive algorithms that identify the functional effect of a variant in the genome. Both classes of variants (SNPs and indels) were further categorized into ‘true’ and ‘not listed’. The latter related to variants that have not appeared or been annotated in dbSNP 138^40^. The ClinVar database which incorporates entries from the OMIM database was used to determine the clinical significance, disease associations and linked phenotypes of the variants that were discovered. VCF miner^65^, a graphical user interface was used for sorting, filtering and querying information encoded in the VCF files. Furthermore, data files containing comprehensive information for centromeres, telomeres, short arms, segmental duplications, and repeats from UCSC Table Browser^41^ were obtained. The repeat dataset was based on RepeatMasker^41^ which comprised a comprehensive set of repeat classes, including SINE (1,793,723), LINE (1,498,690), LTR (717,656), DNA repeats (461,751), simple repeats (417,913), low complexity regions (371,543), various RNA repeats (11,707), satellites (9,566), and others. Note that this in particular included repeat families like 202 *Alu* families (part of the SINE repeat class), 310 L1 families and 115 L2 families (part of the LINE repeat class) and six SVA families (3,733 in total under repeat class ‘other’).

A filter for variants in these regions was applied in Python using an efficient interval-tree data structure.

### Analyses of Y-chromosome and mitochondrial haplogroups

The Y-chromosome variants were called using yHaplo^66^ and Amy-Tree^67^ to construct the haplogroup of the male participant (UAE S001). The default settings of the respective tools were used and followed with the VQSR-filtered SNP set of the recalibrated VCF file, which locates a male based on lineage defining marker SNPs in a top down manner.

The paired-end reads generated for the two samples were previously aligned to the reference, hg19. For the mitochondrial analyses, this lineage sequence was realigned and mapped to the revised Cambridge Reference Sequence (rCRS)^68^. The Haplogrep tool^69^ was used to call the mtDNA Haplotypes.

### Genetic ancestry

For the purpose of defining the genetic ancestry of the UAE population, a cohort of 1,192 citizens of the country were genotyped using the Illumina Omni 5 Exome bead chip (Illumina Inc, San Diego, California, USA). The bead chip contains 4.6 million Single Nucleotide Polymorphism (SNPs), and genotyping was part of a long running project to establish an Emirates Family Registry for anthropological and disease association studies^70^. The genotype data of these Emiratis were compared with the genotype data from the Human Genome Diversity Project (HGDP) using multidimensional scaling (MDS), a form of Principal Components Analysis (PCA). MDS was performed using the PLINK^71^, i.e. SNPs that fail Hardy-Weinberg-Equilibrium test with significance of 0.001, minor allele frequency <1%, missingness <1%. This yielded a data set with 493 K SNPs for all samples. Subsequently, the principal components for UAE S001 and UAE S002 were plotted using Python^72^ and Matplotlib^73^.

### Validation of SNP calls

The Illumina Omni 5 Exome bead chip used for the genetic ancestry was reused for the concordance calculations. The variant calling file (VCF) generated after the recalibration steps for UAE S001 was converted to Plink’s ped/map file format using vcftools^74^. The final comparison between the two sets was performed with a custom Python script concordance, that was used to account for deviations from the reference genome (hg19)^33,34^ and multiallelic loci using dbSNP 138^40^.

### Calculation of intergenome distance between two samples’ genomes and genomes from world populations

In order to contextualize the genomes of UAE S001 and UAE S002 in a comprehensive phylogenetic tree, their variants were compared against subjects from all world populations sampled during the Human Genome Diversity Project (HGDP)^75^ and available data from a neighboring country an individual of similar south/central Asian ancestry from, Kuwait. Due to the comparatively small variant set of the intersection dataset, the final overlap of variants was 20,658. Subsequently all mutual intergenome distances were calculated using Plink’s Identity by state distance measure, which expresses distances as genomic proportions. The resulting distance matrix was subjected to Neighbor Joining using BioPython’s Phylo module^76^. The phylogenetic tree was visualized using iToL2^77^.

## Supplementary information

Table S1

Table S2

## Data Availability

Genome data has been deposited at the European Genome-phenome Archive (EGA) which is hosted at the EBI and the CRG, under Accession Numbers EGAS00001003742 and EGAD00001005119.
